# Electrochemical DNA Sensor Based on Carbon Black—Poly(Neutral Red) Composite for Detection of Oxidative DNA Damage

**DOI:** 10.3390/s18103489

**Published:** 2018-10-16

**Authors:** Yurii Kuzin, Dominika Kappo, Anna Porfireva, Dmitry Shurpik, Ivan Stoikov, Gennady Evtugyn, Tibor Hianik

**Affiliations:** 1 A.M. Butlerov’ Chemistry Institute of Kazan Federal University, 18 Kremlevskaya Street, Kazan 420008, Russia; yzinkyra@mail.ru (Y.K.); almija@mail.ru (D.K.); porfireva-a@inbox.ru (A.P.); dnshurpik@mail.ru (D.S.); Ivan.Stoikov@mail.ru (I.S.); 2 Department of Nuclear Physics and Biophysics, Comenius University, Mlynska dolina F1, 842 48 Bratislava, Slovakia

**Keywords:** DNA sensor, oxidative DNA damage, electropolymerization, poly(Neutral Red), electrochemical impedance spectroscopy

## Abstract

Voltammetric DNA sensor has been proposed on the platform of glassy carbon electrode covered with carbon black with adsorbed pillar[5]arene molecules. Electropolymerization of Neutral Red performed in the presence of native or oxidatively damaged DNA resulted in formation of hybrid material which activity depended on the DNA conditions. The assembling of the surface layer was confirmed by scanning electron microscopy and electrochemical impedance spectroscopy. The influence of DNA and pillar[5]arene on redox activity of polymeric dye was investigated and a significant increase of the peak currents was found for DNA damaged by reactive oxygen species generated by Cu^2+^/H_2_O_2_ mixture. Pillar[5]arene improves the electron exchange conditions and increases the response and its reproducibility. The applicability of the DNA sensor developed was shown on the example of ascorbic acid as antioxidant. It decreases the current in the concentration range from 1.0 μM to 1.0 mM. The possibility to detect antioxidant activity was qualitatively confirmed by testing tera infusion. The DNA sensor developed can find application in testing of carcinogenic species and searching for new antitumor drugs.

## 1. Introduction

DNA and synthetic oligonucleotides (aptamers) have found increasing attention as biocomponents of electrochemical biosensors during the past decades. First and foremost, this is related to the importance of biochemical functions of DNA molecules in living organisms and its potential application in the development of biosensors for detection of pathogens [1,2,3] and biomarkers of various diseases [4,5,6]. Meanwhile, the detection of low molecular species like anticancer drugs [7,8,9], environmental pollutants [10,11,12] and reactive oxygen species (ROS) [13,14,15] are also in the focus of researchers. More than half of the articles on biosensors recently published were focused on the electrochemical biosensors offering undisputable advantages over other portable analytical devices, such as time and labor consuming measurement protocols, high sensitivity, compatibility with conventional measurement instrumentation, possibilities for use in field and automation prospects.

The ROS are radical products of some natural and anthropogenic reactions with molecular oxygen. They are involved in many biochemical paths with participation of biopolymers and are considered as very dangerous for human health because of potential carcinogenicity of the products formed. The ROS are also released in natural enzymatic reactions, e.g., in the cell breathing process in mitochondria [16]. However, the levels are dramatically increased in so-called oxidative stress [17] and in the presence of certain pollutants [18] and ionizing radiation [19]. Interaction of the ROS with DNA results in oxidation of nucleobases, DNA unwinding and primary sequence breaks [20]. Electrochemical detection of oxidative DNA damage can be based on recording of own voltammetric signals attributed to the DNA bases (mostly guanine and adenine [21]). The increase of the appropriate signals results from higher accessibility of the bases for electron exchange caused by disturbances in the DNA steric structure caused by the ROS attack. Besides, new signals attributed to 8-oxoguanine as a main biomarker of oxidative stress appear on voltammograms [22]. To decrease working potential and amplify voltammetric signals, redox probes capable of specific interactions with double-stranded (ds-) DNA, e.g., Co phenanthroline [23], Ru and Co bipyridine complexes [24], or Methylene blue [25] are added to the sample. They are implemented in the ds-DNA structure (intercalation) or coordinate at the surface of the DNA helix [26]. Depending on the details of interactions, the redox probe can be accumulated near the electrode or can lose its redox activity due to steric limitation of the electron exchange within the DNA helix. Appropriate changes of the signal depend on the DNA/probe quantities and degree of the DNA structure damage.

Polymers applied in the assembly of electrochemical DNA sensors are mostly intended for the immobilization of DNA (aptamer) on the electrode surface [27,28,29]. Nevertheless, they were also utilized as heterogeneous redox probes in DNA sensors for detection of hybridization events [30,31]. This made it possible to reduce the number of stages and necessity in additional reagents in the signal measurement protocol and stabilize biosensor signal due to exclusion of diffusional losses of redox probes from the sensing layer. Similar goals are solved by the introduction of modern nanomaterials, e.g., Au nanoparticles and carbon nanotubes that increase specific surface area, decrease the interface resistance, and improve conditions for biopolymer immobilization [32,33,34]. Hence, the combination of electroconductive nanomaterials and electropolymerized coatings working as redox probes for the DNA signal generation is a promising direction for the further progress in DNA sensors development for sensitive detection of DNA oxidative damage.

Previously we have shown the prospects of a new macrocyclic compound, pillar[5]arene (P[5]A), as a redox probe for sensitive determination of oxidizable species [35]. Its decacarboxylated derivative was also applied as an anchor for immobilization of the aptamer against aflatoxin M1 [36]. Own redox activity of P[5]A adsorbed on the carbon black (CB) layer was successfully used for the detection of specific DNA interactions [37]. In this work, we propose a new hybrid coating consisted of P[5]A implemented in the electropolymerized layer of poly(Neutral Red) (poly-NR) on the CB particles for the detection of oxidative DNA damage.

## 2. Materials and Methods

### 2.1. Reagents

P[5]A (1) was synthesized at the Organic Chemistry Department of Kazan Federal University, as described elsewhere [38].

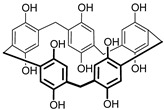
(1)

3-Amino-7-dimethylamino-2-methylphenazine hydrochloride (Neutral Red, NR), ascorbic acid, low molecular weight chitosan were purchased from Sigma Aldrich (Schnelldorf, Germany), low molecular ds-DNA from salmon sperm from Fluka (Schnelldorf, Germany), CB from IMERYS (Willebroek, Belgium). Its structure and purity were confirmed by NMR ^1^H and MALDI TOF mass spectroscopy and elemental analysis. All the other reagents were of analytical grade and used without additional purification.

### 2.2. Oxidative DNA Treatment

Oxidative DNA damage was performed by dissolution of 1 mg of DNA from salmon sperm in 0.9 mL of 25 mM KCl followed by addition of 0.1 mL of 4 mM CuSO_4_. After that, 1.3 μL of 30% H_2_O_2_ were added and the mixture was left for 1 h. In the experiments with ascorbic acid as a model antioxidant, the oxidation mixture was first mixed with ascorbic acid and then added to the DNA solution as described above.

### 2.3. Voltammetric Measurements

Voltammetric measurements were conducted with the CHI Electrochemical Workstation 660E (CH Instruments, Inc., Austin, TX, USA). Glassy carbon electrode made of the rod (geometric surface area 1.67 mm^2^) implemented in the tube of polytetrafluoroethylene with a steel conductor was used as a working electrode for DNA immobilization. Pt wire (CHI 129, CH Instruments, Inc. was used as auxiliary electrode and Ag/AgCl (1 М KCl) (CHI 128, CH Instruments, Inc.) as the reference electrode. All the electrochemical measurements were performed in 25 mM phosphate buffer solution containing 0.1 M NaNO_3_. Electropolymerization was performed by multiple cycling of the potential in 0.4 mM NR buffered solution.

### 2.4. Piezometric Measurements

Piezometric electrochemical quartz crystal microbalance (EQCM) measurements were performed with EQCM module of CHI 440B (CH Instruments, Inc.). All the EQCM experiments were performed in three-electrode non-thermostated cell at ambient temperature. Thin-layer Au electrode of the EQCM chip (basic frequency 8 MHz, 0.205 cm^2^) was used as working electrode. Measurements in continuous flow regime were performed with the EQCM flow cell kit (ALS, cat. No. 012026, Tokyo, Japan) and two-channels syringe pump (ALS, Model 100, cat. No. 012085). The quartz oscillation frequency changes with the mass deposited on its surface, as it is described by the Sauerbrey Equation (2) [39].

(2) $$\Delta f=-\frac{2f_{0}^{2}\Delta m}{A\sqrt{\rho_{q}\mu_{q}}}=-C_{f}\Delta m$$

Here Δ*f* is the frequency shift, *f*_0_ is basic quartz oscillation frequency, Δ*m* is the surface mass change, *A* is the electrode area (0.196 cm^2^), μ is the shear modulus (2.95 × 10^11^ dyn/cm^2^) and *ρ_q_* is the quartz density (2.65 g/cm^3^). The coefficient *C_f_* ascribes sensitivity of the QCM chip to the mass loading. While the application of the Sauerbrey equation is limited by measurements from gaseous media, the conclusion on the frequency shift proportional to Δ*m* can be extended to liquid interface.

### 2.5. Impedance and Scanning Electron Microscopy Measurements

Impedimetric (EIS) measurements were performed with FRA module of galvanostat-potentiostat AUTOLAB PGSTAT 302N (Metrohm Autolab b.v., Herisau, Switzerland) in the presence of 0.01 M K_3_[Fe(CN)_6_] and 0.01 M K_4_[Fe(CN)_6_]. The amplitude of the applied sine potential was 5 mV and the frequency varied from 100 kHz to 0.04 Hz with a sampling rate of 12 points per decade. Capacity and charge transfer resistance values were calculated from the Nyquist diagram using fitting protocol of the NOVA software for C1(R1)-C2(R2) equivalent circuit.

Scanning electron microscopy (SEM) images of the electrode coatings were obtained with the high-resolution field emission scanning electron microscope Merlin™ (Carl Zeiss, Oberkochen, Germany).

### 2.6. Electrode Modification

Modification of glassy carbon electrode with CB and P[5]A was performed as follows. The electrode was first mechanically polished to mirror like surface, treated with acetone, 0.1 M NaOH and H_2_SO_4_ and then electrochemically cleaned by repeated cycling the potential in working buffer solution until minimal background current was reached. After that, it was washed and fixed upside down. Dispersion was prepared by ultrasonication of 1 mg/mL CB in 0.375% chitosan in 0.05 M HCl during 2 h. Then, 1 μL of the dispersion was mixed with 1 μL of 0.1 M NaOH and placed on the surface of the electrode to dry for 20–30 min at 60 °C. After that, 2 μL of freshly prepared 0.1 mM P[5]A solution was spread on the surface and left to dry at ambient temperature. The electropolymerization of NR on the CB/P[5]A layer was performed using the same parameters as those utilized for the experiments with a bare glassy carbon electrode.

## 3. Results

### 3.1. DNA Immobilization

The immobilization of DNA is of critical importance for the detection of specific interactions with low molecular compounds including ROS. The immobilization protocol should provide both accessibility of the biopolymer to the low-molecular agents and stability of the biosensing layer in signal measurement conditions. Among many other methods elaborated for DNA sensor assembling, implementation in the growing polymer film was chosen in this work because of a simple protocol and well reproducible characteristics of the DNA-polymer materials formed.

**Figure 1:**
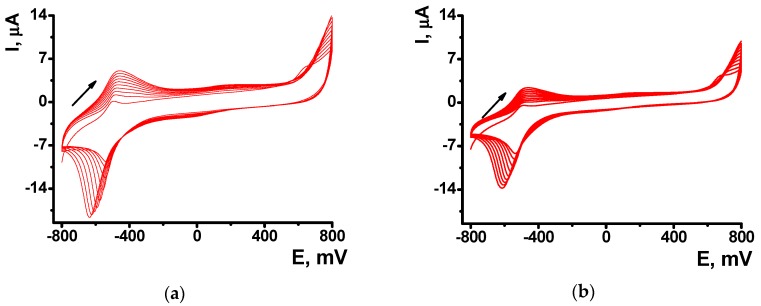
NR electropolymerization by multiple cycling of the potential in 0.025 M phosphate buffer + 0.1 M NaNO_3_ (pH 6.0) containing 0.4 mM NR (**a**) and 0.4 mM NR + 0.2 mg/mL DNA (**b**). Scan rate was 50 mV/s. Arrow shows the direction of the potential scanning.

The polymerization of NR was performed by multiple cycling of the potential within −0.8 and 0.8 V corresponding to the redox activity of the dye. In this potential window, a pair of redox peaks at −0.455 and −0.635 V is attributed to the reversible oxidation (3) of the phenazine moiety whereas irreversible peak at higher anodic potential initiates polymerization [40] (Figure 1).

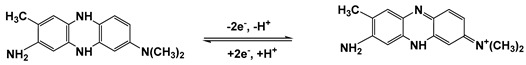
(3)

As the polymeric and monomeric forms have the same redox activity, the peak pair retains its position with increasing number of potential cycles. Their height increased with the number of the cycle, indicating increased amounts of redox active product on the electrode interface. The peak potential difference regularly increases due to limited rate of electron transfer within the film. The addition of DNA to the monomer solution did not alter, significantly, the shape of the peaks on voltammogram and decreased the peak currents due to partial suppression of the polymerization.

**Figure 2:**
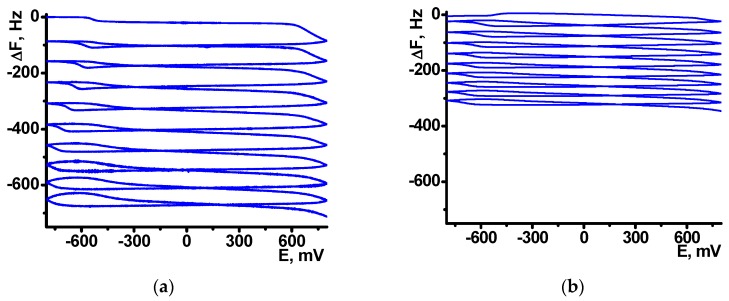
The electrochemical quartz crystal microbalance sensograms recorded on Au electrodes of quartz crystal chip during the multiple cycling of the potential in the 0.4 mM NR solution in 0.025 M phosphate buffer + 0.1 M NaNO_3_ (рН = 6.0) in the absence (**a**) and in the presence of 0.2 mg/mL DNA (**b**).

The formation of polymeric form was confirmed by EQCM. In this method, electropolymerization is performed on golden film electrodes deposited on the surface of the AT cut of quartz crystal disk. Appropriate curves are presented in Figure 2 for the NR electropolymerization performed in the absence and in the presence of 0.2 mg/mL DNA.

Absolute frequency shift and appropriate *C_f_* values were twofold lower in the presence of DNA. Probably, non-conducting biopolymer molecules are entrapped in the poly-NR film and disturb electron exchange in the film necessary for polymerization. Besides, they can partially block the surface of the electrodes. Changes in the morphology of the curves should be also mentioned. IT is well known that cycling the potential results in changes of the parameters of redox active polymers like reversible inclusion of counter ions, release of low molecular by-products of polymerization etc. [41]. As a result, a sharp sift of the mass is observed in the area of −600 mV corresponded to the redox reaction and counter ion involvement. In the presence of DNA such changes are much milder because DNA is a less mobile molecule, which is held in the polymer layer contrary to low molecular ions of indifferent electrolyte. The above changes on voltammograms and sensograms, caused by DNA addition increase with the quantities of the biopolymer added.

**Figure 3:**
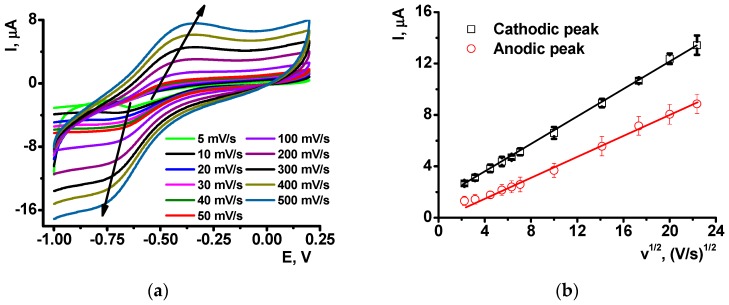
Cyclic voltammograms recorded on glassy carbon electrode covered with poly-NR-DNA coating (**a**) and dependence of the cathodic and anodic peak currents on the square root from the scan rate (**b**). Results are mean ± SD from six repetitions.

After the NR electropolymerization, the electrodes were transferred in the working buffer with no monomeric dye and the redox activity of the coating was assessed using direct current voltammetry. As on the electropolymerization stage, one reversible peak was found in the same peak potentials corresponded to the poly-NR redox activity. The peak currents changed with the potential scan rate in accordance with formal diffusional limitation of the electron transfer (Figure 3). As no monomers were contained in the solution, diffusional limitation can be referred to the shuttle mechanism of the electron exchange between reduced and oxidized NR units in the polymer chain.

**Figure 4:**
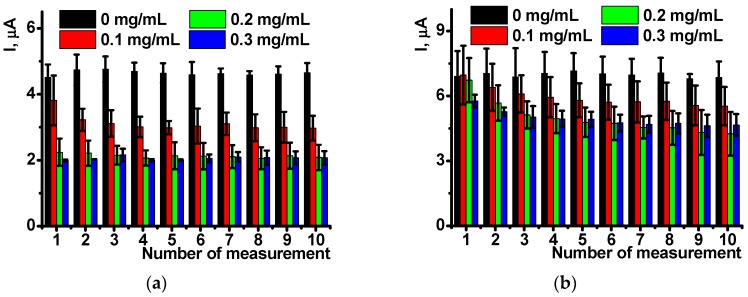
The reproducibility of the oxidation (**a**) and reduction (**b**) peak currents recorded on glassy carbon electrode modified with poly-NR layer obtained in the presence of various amounts of DNA. Scan rate 50 mV/s. Results are mean ± SD from 5 repetitions.

The peaks on votlammograms were quite stable in aqueous solutions. The shape and height of the peaks retain during the working day without significant changes. Figure 4 shows the appropriate dependencies for ten consecutive measurements performed on the same sensor with intermediate stirring of the working solution in open circuit mode. The results are averaged for five sensors.

As follows from the Figure 4, the influence of DNA present in polymerization mixture is pronounced at its low concentration and is more expressed in oxidation currents. Reduction peaks are less reproducible and stabilized to the 3rd–4th measurement. This might be due to higher impact of negatively charged DNA molecules on the positively charged poly-NR chain and hence rearrangement of the film structure during first cycles of the potential.

### 3.2. Influence of pillar[5]arene on the DNA Sensor Performance

**Figure 5:**
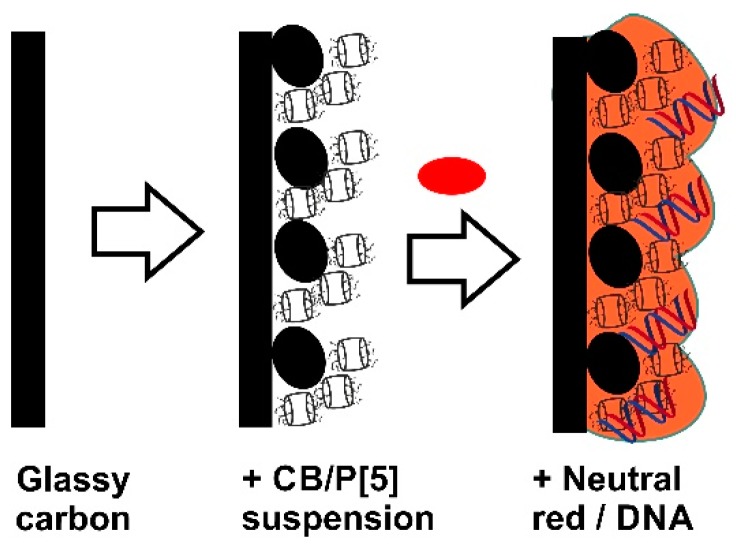
Schematic outline of the deposition of the components and formation of the surface layer by the NR polymerization in the presence of DNA.

Mediators of electron transfer improve the electron exchange reactions on the electrode interface and promote determination of redox conversion of the analytes and DNA specific agents [42]. Previously it was shown that P[5]A, a macrocyclic compound consisted of hydroquinone units bonded with methylene bridges, can be used in electrochemical sensors due to high sensitivity of redox reactions to the external factors. Thus, DNA promotes the P[5]A redox activity by disturbance of hydrogen bonds between neighboring hydroxyl groups of the macrocycle moiety [37]. Direct adsorption of P[5]A on glassy carbon was found inefficient because its oxidation products suppressed the amperometric signal [35]. For this reason, the electrode was first modified with stabilized adsorbed macrocycle and allowed multiple measurement of its redox activity. The modification was performed by placing of the CB suspended in chitosan and following addition of P[5]A. No significant difference in biosensor performance was found if P[5]A was preliminary adsorbed on the CB followed by deposition of suspension on the electrode or its solution was stepwise added to the GCE covered with the CB particles. As a result, a swell reproducible coating was formed as support for the NR electropolymerization. The protocol of surface layer assembling is schematically outlined in Figure 5.

**Figure 6:**
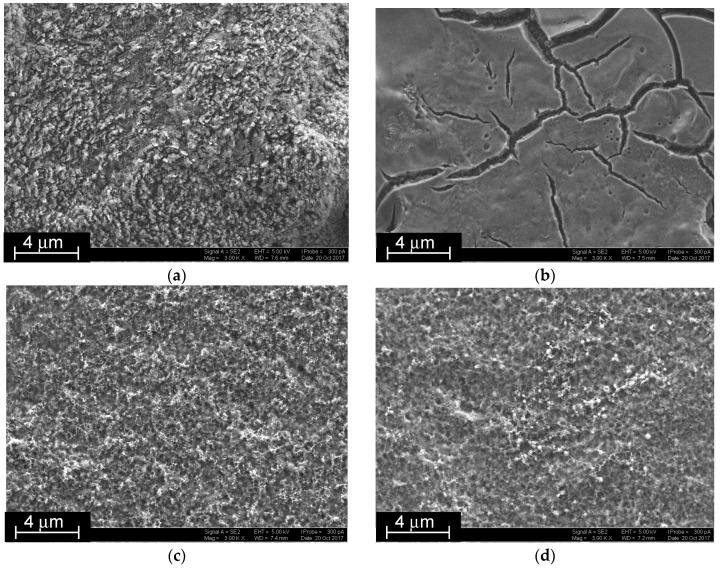
SEM images of glassy carbon covered with CB (**a**), that after P[5]A deposition (**b**), CB/P[5]A/poly-NR film (**c**) and that obtained in the presence of 0.2 mg/mL of DNA (**d**).

The CB suspension made impossible EQCM characterization of the assembling due to overloading of the QCM chip surface. Instead, the SEM images were obtained on various stages of surface layer assembling (Figure 6). Just after the CB loading, a tough surface with round elongated articles partially embedded in the chitosan matrix was obtained (Figure 6a). P[5]A smoothened the surface with formation of vitreous film with rather deep cracks. This was attributed to the association of the macrocycles to supramolecular polymers via extramolecular hydrogen bonds between hydroxyl groups of the macrocycle (Figure 6b). The electropolymerization of the NR, taken either alone (Figure 6c) or together with DNA (Figure 6d), destroyed the P[5]A association and resulted in the deposition of regular fungous coating with mesopores evenly distributed along the surface.

**Figure 7:**
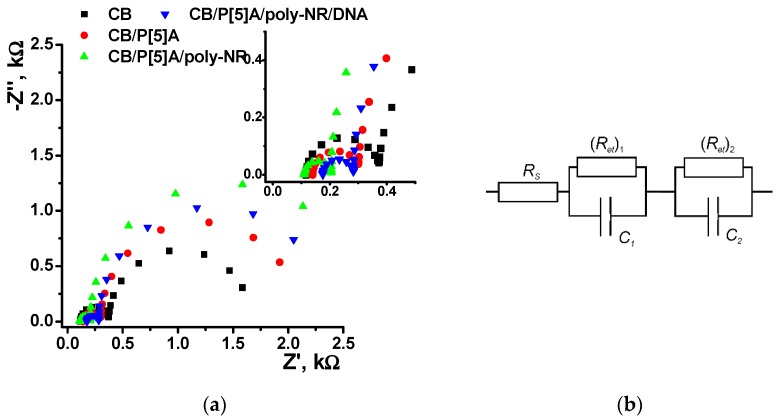
(**a**) The Nyquist diagram of impedance spectra recorded with glassy carbon electrode covered with CB, CB/P[5]A, CB/P[5]A/poly-NR and CB/P[5]A/poly-NR/DNA layers (Inset: small semicircle area). Measurements in the presence of 0.01 M K_3_[Fe(CN)_6_] and 0.01 M K_4_[Fe(CN)_6_] at 220 mV vs. Ag/AgCl. Frequency range 0.04 Hz–100 kHz, amplitude 5 mV. (**b**) Equivalent circuit: *R_S_*—solution resistance, *R_et_*—charge transfer resistance, *C*—capacitance, 1 and 2 indices correspond to the inner and outer interface of the layer.

Assembling of the layers on the glassy carbon surface was also confirmed by EIS with ferricyanide redox probe (Figure 7). The Nyquist diagram contained two semicircle parts corresponding to the electron transfer on the inner and outer interfaces of the surface layer as limiting steps of electron exchange. The EIS parameters obtained from the Nyquist diagram by data fitting are summarized in Table 1. The roughness coefficient (*n* = 0.89–0.92) corresponded to nearly ideal behavior of the system and hence corresponded to its interpretation as capacitance of the interface. On the layer/solution interface, the charge transfer resistance increased with the addition of the P[5]A and then after the NR polymerization due to rather slow electron exchange through the films of the above components. The addition of DNA in reaction mixture decreased the *R_et_* value though the DNA itself is neither electroconductive nor electrochemically active. Probably this is a result of the templating effect of DNA. It promotes the polymerization and stabilizes positively charged oxidized form of the poly-NR. Its changes during the surface layer assembling were opposite to those of charge transfer resistance and coincided well with alterations in the charge separation. Thus, addition of P[5]A suppressed the dissociation of carboxylic groups of CB particles and partially blocked their surface and hence reduced the capacitance. The NR electropolymerization increased this parameter because of the positive charge of the poly-NR layer formed. The same process conducted in the presence of negatively charged DNA slightly decreased maximal capacitance. In this case, the neutralization of the polymer positive charge is partially compensated for by higher quantities of the poly-NR, where deposition is stimulated due to template DNA influence.

Contrary to that, the EIS parameters on the electrode/layer interface remain about constant in all the steps of the assembling.

**Figure 8:**
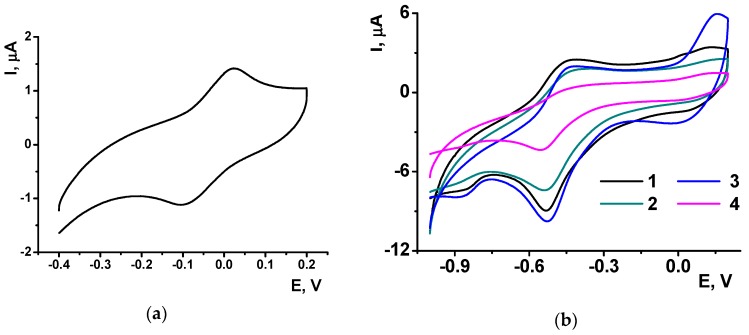
Cyclic voltammogram recorded on glassy carbon electrode covered with CB (2 μL of 0.1 mg/mL suspension per electrode) and P[5]A (2 μL of 0.1 mM solution per electrode) (**a**); Cyclic voltammograms of the poly-NR recorded in the absence of the monomer dye, the CB/P[5]A layer was obtained with 0.05 (1), 0.1 (2), 0.5 (3) and 1.0 (4) mM P[5]A in the aliquot of 2 μL (**b**). All the measurements in 0.025 M phosphate buffer, pH 7.0. Scan rate: 50 mV/s.

P[5]A gives on the glassy carbon electrode covered with CB a symmetric peak pair at −0.1,…, 0 V (Figure 8a), which remains constant in multiple cycling of the potential in the NR solution. In resulting voltammogram recorded during the electropolymerization in the NR solution, the P[5]A signal is suppressed against that of the NR though its position on the potential axe remains the same as on bare electrode covered with CB.

The poly-NR layer obtained on the CB/P[5]A coating shows in buffer solution with no monomer the same reversible peak pair as on bare glassy carbon electrode. Meanwhile the peaks seem less widen and more symmetrical than those recorded on bare electrode (Figure 8b). The NR peak currents remain about the same at low concentrations of the P[5]A, whereas high concentration of the macrocycle decreases the NR peaks probably due to partial blocking of the electrode with by-products of the P[5]A oxidation. The mechanism of signal decay is confirmed by the increase of the P[5]A influence with the number of potential cycling. Thus, the use of P[5]A improves the conditions of the electron exchange in the poly-NR layer.

### 3.3. Detection of Oxidative DNA Damage

Oxidation of native DNA is a part of oxidative stress initiated by radical reactions with so-called reactive oxygen species (ROS) formed both in endogenous and exogenous reactions [43]. Increased release of ROS can increase the probability of oncogenesis and suppress immunity of a human being. Electrochemical DNA sensors developed for DNA damage detection can be considered as early warning devices signaling on dangerous contamination of the foodstuffs and environment with oncogenes. Even though natural processes of DNA repair are not taken into account, such biosensors are useful for establishing of safer comfortable life conditions.

In this work, the DNA damaging factor was modeled by Cu^2+^/H_2_O_2_ mixture generating hydroxyl radicals in accordance with (4) [44]. All the experiments were performed with 0.2 mg/mL DNA solution provided the most reproducible results.

2Cu^2+^ + H_2_O_2_ → 2Cu^+^ + O_2_ + 2H^+^ Cu^+^ + H_2_O_2_ → Cu^2+^ + OH^−^ +∙OH (4)

EQCM measurements showed the influence of oxidized DNA on the NR polymerization comparable to that of native DNA (Figure 8a). The mass shift was lower in the presence of oxidized a DNA due to lower effective area of the electrode participation in the electron transfer and hence polymerization and deposition of appropriate products. The comparison of the native and oxidized DNA did not show significant difference in the resulting Δ*F* value.

**Figure 9:**
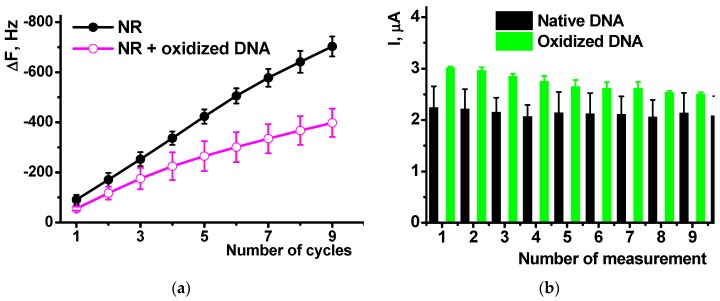
The influence of DNA oxidation on the efficiency of the NR polymerization monitored by EQCM (**a**) and on the poly-NR cathodic peak current (**b**) recorded by cyclic voltammetry on bare glassy carbon covered with poly-NR. DNA 0.2 mg/mL, measurements in 25 mM phosphate buffer + 0.1 NaNO_3_, pH 6.0. Results are mean ± SD from six replications.

Cyclic voltammograms offer similar sensitivity of the NR peak currents to the DNA oxidation. Thus, the NR peaks recorded after polymerization of the dye on bare glassy carbon in the presence of oxidized DNA were about 15% higher than those obtained with native DNA in polymerization solution. This might be due to higher flexibility and partial unwinding of the damaged DNA molecules that can form more dense complexes with growing polymer chains and hence stabilize oxidized form of poly-NR. Meanwhile the difference between them (Figure 9b) tends to decrease in the series of consecutive measurements form the same solution. This can be referred to either partial leaching damaged DNA fragments from the surface layer or rearrangement of the film structure in several oxidation-reduction cycles.

The influence of P[5]A in the surface layer on the detection of the DNA damage was first considered using the EIS technique. The oxidation of DNA diminished its influence on the charge transfer resistance recorded in the presence of ferricyanide redox probe. Thus, on the layer/solution interface the *R_et_* decreases from 2.4 (poly-NR) to 2.1 kΩ against 1.8 kΩ for the coating with native DNA (all the experiments with 0.2 mg/mL DNA in polymerization solution). The capacitance increases to 650 μF, a medium value between that of poly-NR with no DNA (876 μF) and poly-NR/DNA (576 kΩ). All the changes are insignificant from the point of view of DNA damage detection because they are comparable with the 10% deviation of the EIS parameters.

**Figure 10:**
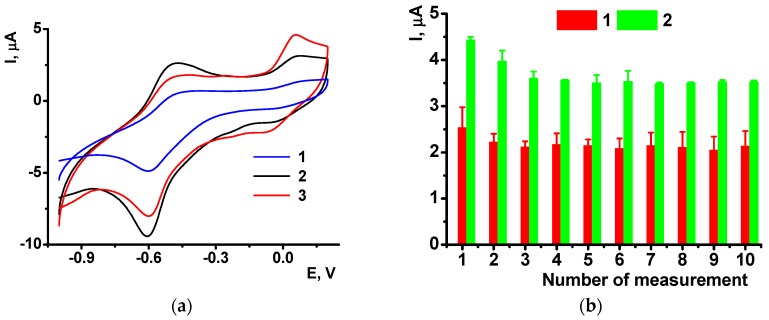
The poly-NR peaks recorded on glassy carbon electrode covered with CB/P[5]A in the absence of the monomer dye, the NR polymerization was performed in the presence of native (1), oxidized (2) DNA and that oxidized in the presence of 1.0 mM ascorbic acid (3) (**a**). Changes in the oxidation peak currents recorded in the presence of native (1) and oxidized (2) DNA in the series of consecutive measurements performed in 0.025 M phosphate buffer, pH 7.0 (**b**). Scan rate: 50 mV/s. Results are mean ± SD from six replications.

The cyclic voltammograms showed significant increase in the peaks recorded in the presence of oxidized DNA (Figure 10a). The signal is quite stable and stabilizes to third measurement performed in the same solution (Figure 10b). Relative drift of the signal is similar to that obtained with native DNA in the polymer layer. The reproducibility of the peak current is paradoxically increased with DNA oxidation: the R.S.D. of 4.2% and 2.3% were obtained for six individual sensors containing poly-NE with native and damaged DNA, correspondingly.

The influence of DNA oxidation on the biosensor behavior was confirmed by modeling experiments with ascorbic acid as an antioxidant. The addition of 1.0 μM–1.0 mM ascorbic acid to the oxidation mixture decreased the influence of oxidation as shown in Figure 9a for 0.1 mM ascorbic acid as an example. This is due to protective effect, i.e., interaction of ascorbic acid with free radical particles generated in Cu^2+^/H_2_O_2_ mixture. The influence of the antioxidant was quantified by calibration Equation (5) expressing the decrease of the DNA damage as a function of ascorbic acid concentration. The absolute cathodic current of poly-NR was chosen as a measure of DNA conditions in the surfaced layer.

*I*, μA = (6.90 ± 0.12) + (0.96 ± 0.03) × (*C*, mM), *n* = 5, R^2^ = 0.9565 (5)

### 3.4. Measurement Precision and DNA Sensor Lifetime

As was mentioned above, measurement precision was estimated in two experimental series, i.e., for an individual sensor in ten consecutive measurements performed from the same working buffer with intermediate stirring of the solution in open circuit mode, and for six individual sensors prepared from the same set of reagents. The measurement-to-measurement repeatability calculated for the DNA sensor with polymer layer deposited on bare glassy carbon electrode was equal to 3.2% and that of the sensors based on PB/P[5]A layer to 4.2%. Sensor-to-sensor repeatability of the same series was equal to 4.3 and 5.0%. Substitution of native DNA with that treatment with Cu^2+^/H_2_O_2_ decreased the R.S.D. probably due to more dense and reproducible assembly of the sensor layer.

The DNA sensor stored in dry conditions at 4 °C retains its voltammetric response for at least three months. For this period of time, the cathodic peak current of poly-NR decreases by 20% of the initial value.

## 4. Discussion

The polymerization of NR in the presence of DNA added to the monomer solution prior to potential cycling resulted in implementation of the biopolymer molecules in the growing film of polymeric dye on the electrode. Similarly to phenothiazine dyes investigated earlier [41], the presence of DNA affects the equilibrium of redox conversion of the polymeric dye on the electrode. Contrary to polyaniline, this response can be monitored in neutral media. This makes the hybrid material proposed very attractive for preliminary control of cancerogenic factors and of reactive oxygen species. Preliminary oxidation of DNA prior to its introduction in the biosensor assembly is more reliable than alternative probing hazardous samples with DNA sensors [32], where many other factors interfere with detection of the DNA damage. This compensates for complicated measurement protocol assuming assembling surface layer in each measurement.

The use of additional mediator of electron transfer, i.e., pillar[5]arene, improves the conditions of redox reactions and enhances sensitivity of the response toward oxidative DNA damage. The positive influence of P[5]A is expressed in increased reversibility of the surface electrode reactions resulted from the involvement of the macrocycle in the electron transfer chain. Besides, it follows from the series of repeated runs, the P[5]A presence improves measurement-to-measurement repeatability of the results. Then, it stimulates changes, referred to as DNA damage due to the mutual influence of DNA and P[5]A on the redox activity of the macrocycle as was shown recently in our work concerning detection of specific DNA interaction by changes in the P[5]A redox activity [37]. It was shown that DNA can enhance the redox activity of pillar[5]arene by suppression intermolecular interactions via hydroxyl groups of the macrocycle moiety. As a result, the signal toward damaged DNA and its repeatability increased twice. Such an effect can be used in other applications of hybrid materials obtained, e.g., for the electric transduction of enzymatic reactions or for detection of low molecular compounds able to specific interactions with DNA (anticancer drugs).

The DNA sensor developed does not allow estimating relative degree of the DNA damage. However, it can be assessed from the difference of the peak currents used for its detection. Indeed, the signal is increased about twice. This exceeds the changes observed in some other DNA sensors utilizing parameters of the redox activity as a measure of DNA damage [32,37]. In the future, the DNA sensor proposed can find application in preliminary testing foodstuffs for antioxidant activity or for the determination of potentially cancerogenic contaminations of soil or industrial products contacting with humans.

## Figures and Tables

**Table 1:** The electrochemical impedance spectroscopy parameters obtained for stepwise assembling of the surface layer of DNA sensor. Measurement conditions are presented in legend to Figure 6. Average of six replicates.

Layer Content	(*R_et_*)_1_, Ω	*C*_1_, µF	(*R_et_*)_2_, kΩ	*C*_2_, µF
CB	270 ± 15	1.6 ± 0.1	1.3 ± 0.1	670 ± 30
CB/P[5]A	305 ± 15	2.2 ± 0.1	1.7 ± 0.1	455 ± 25
CB/P[5]A/poly-NR	275 ± 10	1.4 ± 0.1	2.4 ± 0.1	876 ± 40
CB/P[5]A/poly-NR/DNA	327 ± 253	3.6 ± 0.2	1.8 ± 0.1	576 ± 44
